# Diabetes mellitus and renal involvement in chronic viral liver disease

**Published:** 2015

**Authors:** VF Iovanescu, CT Streba, M Ionescu, AF Constantinescu, CC Vere, I Rogoveanu, E Moța

**Affiliations:** * University of Medicine and Pharmacy of Craiova, Craiova, Romania; ** Gastroenterology Department, University of Medicine and Pharmacy of Craiova, Craiova, Romania; *** Department of Medical Informatics and Biostatistics, University of Medicine and Pharmacy of Craiova, Craiova, Romania; **** Department of Nephrology, University of Medicine and Pharmacy of Craiova, Craiova, Romania

**Keywords:** chronic viral hepatitis, viral liver cirrhosis, diabetes mellitus, kidney disease, hepatocellular carcinoma

## Abstract

**Hypothesis:** Chronic viral liver disease is often associated with other conditions. Diabetes mellitus (DM) is frequently reported in this context and may play a role in the progression of the liver disease to hepatocellular carcinoma (HCC). Renal disease is also an important extrahepatic manifestation of hepatitis viral infection and its presence is associated with poor prognosis and management issues.

**Objectives:** Our study had multiple purposes: to determine the frequency of the association between chronic viral liver disease and diabetes mellitus, evaluate the potential of diabetes mellitus as a risk factor for HCC and assess an eventual renal involvement.

**Methods and results:** We included in our study a number of 246 patients with chronic liver disease, from whom 136 were diagnosed with chronic viral hepatitis and 110 with viral liver cirrhosis. These patients were assessed by using a clinical examination and a series of tests, including serum transaminase levels, serum bilirubin, serum albumin, markers of cholestasis, fasting plasma glucose levels, serum creatinine, urea, albuminuria, Addis-Hamburger test, electrophoresis of urinary proteins, abdominal ultrasound and, in some cases, CT examination. We obtained the following results: diabetes mellitus is often associated with chronic liver disease of viral etiology, having been identified in 18.29% of the patients in our study. Age above 60 in patients with chronic hepatitis (p=0.013<0.05) and presence of hepatitis C virus were particularly correlated with the presence of diabetes mellitus. Renal disease was present in 13.4% of the patients with chronic liver disease and it was especially associated with liver cirrhosis and hepatitis C virus. The most common form of renal injury was glomerulonephritis. Acute kidney injury was diagnosed only in cirrhotic patients as hepatorenal syndrome, occurring in 7.27% of the subjects, while chronic kidney disease was identified only in two cases of chronic viral hepatitis. Four patients in our study were diagnosed with HCC and none of them presented diabetes mellitus. **Discussion:** Our study revealed that there is a significant association between diabetes mellitus and chronic viral liver disease induced by hepatitis C virus. Glomerulonephritis was the most common type of renal disease in both hepatitis patients and in those with cirrhosis. Glomerular injury was strongly correlated with the presence of hepatitis C virus than with hepatitis B virus. A connection between diabetes mellitus and hepatocellular carcinoma could not be established.

## Introduction

Chronic liver disease, including chronic viral hepatitis and viral liver cirrhosis, are known to be associated with diabetes mellitus (DM) [**1**,**2**]. A connection between chronic viral hepatitis and type II diabetes mellitus was suspected for the first time many years ago, when studies conducted on patients with chronic hepatitis B and C revealed the presence of diabetes mellitus in a significant number of cases. Since then, numerous trials have augmented the belief that the two entities are indeed related, although the exactly underlying pathophysiological mechanisms have not yet been established. There is a significant difference regarding the frequency of type II diabetes mellitus in different types of chronic viral hepatitis. This refers to the fact that chronic hepatitis C virus (HCV) infection is more frequently associated with diabetes mellitus, compared to hepatitis B virus (HBV) infection [**3**,**4**]. In liver cirrhosis, diabetes mellitus develops at a significant rate, most of the diabetic cases being related to the alcoholic etiology. Different studies conducted on cirrhotic populations state that the frequency at which it occurs ranges from 10 to 60% [**5**]. The pathogenic mechanisms of this so-called “hepatogenous diabetes” are thought to be related to insulin resistance that develops during the progression of the liver disease [**6**]. The chronic liver disease-diabetes mellitus association may however be a double-edged sword, since some authors claim that the occurrence of diabetes mellitus in cirrhotic patients may accelerate the progression of fibrosis and may lead to hepatocellular carcinoma (HCC) [**7**,**8**].

Renal involvement is an important matter in this context and this is due to several causes. Kidney injury may be related to the chronic liver disease, to the diabetes mellitus, or both [**9**,**10**]. It may occur as an extrahepatic manifestation in the evolution of both chronic viral hepatitis and liver cirrhosis and is associated with poor prognosis. Renal disease in chronic viral hepatitis and liver cirrhosis comprises a wide spectrum. By far, the most often renal manifestation in chronic viral hepatitis is glomerular injury [**11**,**12**]. Different types of glomerulonephritis occur with variable frequency, depending on the type of hepatitis virus that is involved. Acute kidney injury (AKI) is exceptional in chronic viral hepatitis, while chronic kidney disease (CKD) is possible to occur, especially as an evolutive consequence of glomerulonephritis [**13**]. Kidney disease may also occur in the context of liver cirrhosis, either as glomerular injury, or, more often, as hepatorenal syndrome [**14**,**15**]. On the other hand, patients with diabetes mellitus may also develop renal disease, especially after many years of disease progression [**16**,**17**]. Furthermore, the presence of chronic viral hepatitis in patients with diabetes mellitus may have a negative impact on the evolution of the diabetic nephropathy.

## Methods

Our study had multiple purposes: to determine the frequency of the association between chronic viral liver disease and diabetes mellitus, evaluate the potential of diabetes mellitus as a risk factor for HCC and assess an eventual renal involvement.

We conducted a prospective study that included a total of 246 patients diagnosed with chronic viral liver disease, patients who were hospitalized in the Clinical Emergency Hospital of Craiova, Romania, between August 2014 and June 2015. Among these, 136 patients had chronic viral hepatitis, while 110 patients had liver cirrhosis of viral etiology. The demographic characterization of the study group revealed that 148 patients were female and 98 patients were male. Patients were aged between 30 and 87, with a mean age of 60,6 (±9,40) for those diagnosed with hepatitis and 61,13 (±11,2) for the patients with liver cirrhosis. We evaluated these patients by using standard biological and imaging tests available in our center. In order to assess the chronic liver disease, we made use of clinical data, as well as a series of tests such as serum transaminase levels, serum bilirubin, serum albumin, markers of cholestasis, abdominal ultrasound and, in some cases, CT examination. The diagnosis of diabetes mellitus was established by measuring fasting plasma glucose levels, according to WHO guidelines on diabetes mellitus. In consequence, patients with fasting plasma glucose above 126 mg/ dl or 2–h plasma glucose above 200mg/ dl and chronic viral liver disease were included in our study. Kidney function was evaluated in all of the patients in the study group by using a series of tests such as serum creatinine and urea, albuminuria, Addis-Hamburger test and electrophoresis of urinary proteins. eGFR was calculated (estimated glomerular filtration rate) for each patient that had evidence of renal disease. 

Statistical analysis was achieved by using Microsoft Excel and made use of Pivot Tables, Functions-Statistical, Charts and Data Analysis modules. Microsoft Excel was also used in complex statistical analysis, such as the Chi Square test. A p-value <0.05 was considered statistically significant.

Our study complies with the Declaration of Helsinki and was approved by the Ethics Committee of the University of Medicine and Pharmacy of Craiova, Romania. Each patient included in this study signed an informed consent.

## Results

**Fig. 1:**
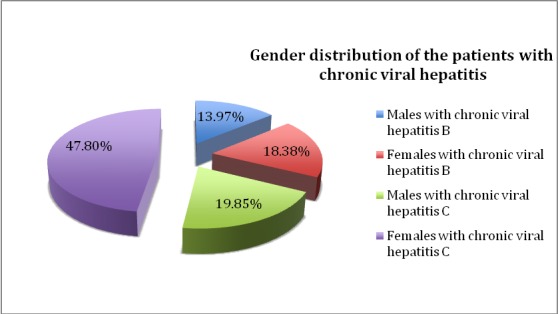
Gender distribution of the patients with chronic viral hepatitis

This study included 246 patients diagnosed with chronic liver disease of viral etiology, aged between 30 and 87, and comprised two groups: 136 patients with chronic viral hepatitis and 110 patients with liver cirrhosis of viral etiology. Within the hepatitis study group, 44 patients, representing 32.35%, had chronic hepatitis B, whilst 92 patients, accounting for 67.65%, were diagnosed with chronic hepatitis C. In this group, females, accounting for 90 patients of the group, represented the majority, while 46 patients were males. The gender distribution of the patients with chronic viral hepatitis is displayed in **Fig. 1**.

**Fig. 2:**
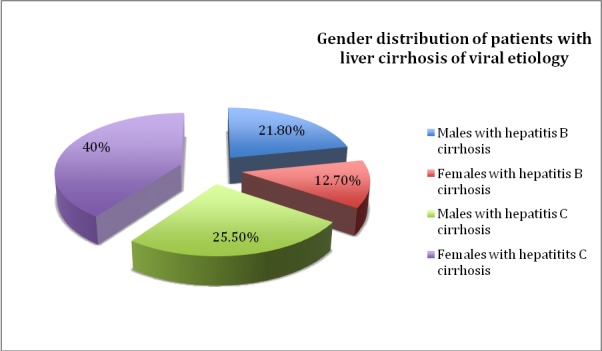
Gender distribution of the patients with liver cirrhosis of viral etiology

Things were very similar in the cirrhosis study group. Most cases of cirrhosis were due to hepatitis C virus, which was responsible for 65,46% of the cases (72 patients). Hepatitis B virus was responsible for 38 cases of cirrhosis (34,54%). The gender distribution of patients with liver cirrhosis of viral etiology is shown in **Fig. 2**. 

Regarding the presence of diabetes mellitus, we discovered that there was a significant association between this entity and chronic liver disease. 45 patients with chronic liver disease of viral etiology were found to have diabetes mellitus at the time of inclusion in the study. This is equivalent for 18.29% of our entire study group. Among these, 22 patients had chronic viral hepatitis, and the other 23 patients came from the cirrhosis study group. These results showed us that diabetes mellitus is a more frequent association in patients with viral liver cirrhosis, having been identified in 20.9% of these cases, in contrast to chronic viral hepatitis, where 16.17% of the patients were found to have this comorbidity, considering the fact that the group with chronic viral hepatitis included more patients than the one with cirrhosis. Our analysis revealed that, in both study groups, diabetes mellitus was more frequently associated with the presence of hepatitis C virus. Thus, in patients with chronic viral hepatitis, 11.76% were diagnosed with hepatitis C and diabetes mellitus and only 4.41% had hepatitis B and diabetes. Likewise, 15.45% of the patients with hepatitis C cirrhosis associated diabetes mellitus, while 5.45% of the hepatitis B cirrhosis were found to have this morbid association. We also took into account the relationship between the patient’s age and diabetes mellitus. Statistical analysis allowed us to identify age as a risk factor for diabetes mellitus in patients with chronic viral hepatitis, but not with liver cirrhosis, based on the results of Chi square test. Patients with chronic viral hepatitis aged above 60 presented a significant risk of developing diabetes mellitus (p=0.013<0.05). In contrast, age above 60 was not identified as a risk factor for the development of diabetes mellitus in viral liver cirrhosis (p=0.543>0.05).

Another clinical parameter we took into account was the presence of hepatocellular carcinoma. Two patients with chronic viral hepatitis and two with liver cirrhosis had evolved towards HCC, although none of them associated diabetes mellitus. As a result, we could not establish a connection between these two conditions. 

A significant incidence of renal disease was found in patients with chronic viral hepatitis and liver cirrhosis. 13.4% of the patients from the entire study group were diagnosed with kidney disease.

As expected, renal disease was more frequently associated with liver cirrhosis than with chronic viral hepatitis, being identified in 17.27% of the cirrhotic patients and in 10.29% of the patients with chronic hepatitis. This fact is due to a high incidence of hepatorenal syndrome in cirrhotic populations. Renal disease in our patients included a spectrum that consisted of glomerular disease, acute kidney injury and chronic kidney disease. Glomerular injury was present in 12 patients with chronic viral hepatitis, representing 8.82% of this group. Four of these patients (2.9%) did not have any other associated condition that could have been responsible for the occurrence of the glomerulopathy, leaving the hepatitis as sole responsible for glomerular injury. Six other patients also had arterial hypertension and two associated both arterial hypertension and diabetes mellitus. 

**Fig. 3:**
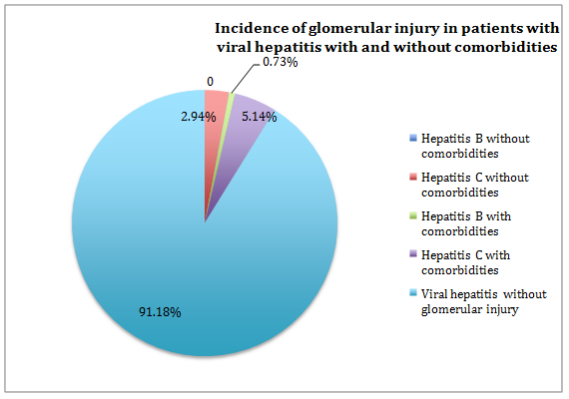
Incidence of glomerular injury in patients with viral hepatitis with and without comorbidities

**Fig. 3** shows the frequency of glomerular injury in patients who also had arterial hypertension, diabetes mellitus or both, in addition to the chronic viral hepatitis in comparison to those who associated only chronic viral hepatitis and glomerulonephritis, correlated with the type of hepatitis virus. As shown, glomerular injury was identified with a higher incidence in patients with chronic hepatitis C in comparison with hepatitis B patients.

Thus, various types of morbid associations of chronic hepatitis B or C present different percentages; however, the result of the Chi square test is greater than the accepted significant value (p=0.199>0.05), mostly due to a relative small number of cases included in each category. The same test was applied to the group of 110 patients with liver cirrhosis of viral etiology and the associated morbidities, resulting a value of p=0.576>0.05.

Viral liver cirrhosis made no exception regarding the presence of glomerulonephritis. 10% of these patients were found to have glomerular injury, although only 1.8% had no other associated condition except for the liver disease that may have induced glomerular injury. Regarding the etiology of the cirrhosis, glomerular injury was especially related to the presence of HCV.

Acute kidney injury was not identified in patients with chronic viral hepatitis, while chronic kidney disease was found in 2 patients, representing 1.47%. Both patients presented signs of glomerular injury, although, being given the fact that one of these patients also had arterial hypertension, and the other presented both arterial hypertension and diabetes mellitus, it is difficult to establish if CKD was a consequence of hepatitis-related glomerulonephritis, arterial hypertension, diabetes mellitus, or a mixture of these factors. AKI is an entity that not rarely occurs in cirrhotic patients. This fact can be due to gastrointestinal bleeding, use of diuretics or occurrence of the hepatorenal syndrome. None of the cirrhotic patients presented arguments for chronic kidney disease although 7.27% had evidence for acute kidney injury. All of these cases fulfilled the criteria for hepatorenal syndrome. 

## Discussions

This study allowed us to uncover a series of important facts. We were able to identify that there is a significant association between chronic viral liver disease and diabetes mellitus. Thus, in a group of 246 patients, a percentage of 18.29% were diagnosed with diabetes mellitus, showing a strong association of the two entities. There are differences regarding the incidence of DM in chronic viral hepatitis and cirrhosis. Our study showed that patients with liver cirrhosis of viral etiology are more prone to developing DM compared to those with viral hepatitis. Further analysis revealed that most patients with chronic viral liver disease that also have diabetes mellitus are infected with hepatitis C virus, raising the question if there exists a specific link between this type of hepatitis virus and diabetes and if hepatitis C virus represents a risk factor for the development of diabetes. Age above 60 was also identified as a risk factor for the occurrence of diabetes mellitus in patients with chronic viral hepatitis (p=0.013<0.05), although not in cirrhosis.

Some authors stated that there is a connection between diabetes mellitus and the progression of the chronic liver disease to hepatocellular carcinoma. In our subjects, CHC was present in four patients, although none of these associated DM. Further studies, conducted on a higher number of patients, where this association can be emphasized, with a longer follow-up period, are necessary in order to correctly establish if these two entities are indeed related.

Renal disease is also an important aspect of chronic liver disease, which we found to be present in 13.4% of our subjects. Most of the patients with kidney disease belonged to the cirrhosis study group. Although kidney damage may take many forms in the context of chronic liver injury, the most frequent type of renal disease that we found was represented by glomerulonephritis. Glomerular injury was the most common type of renal damage both in the hepatitis patients and in those with cirrhosis. Glomerular injury was strongly correlated with the presence of HCV than with HBV, both in patients with chronic viral hepatitis and liver cirrhosis.

Acute kidney injury (AKI) is exceptionally rare in patients with chronic hepatitis and was not identified in any patient with chronic viral hepatitis. On the other hand, chronic kidney disease was found in two patients with evidence of glomerular disease, although both associated conditions that might be responsible for glomerulonephritis, making it difficult to identify the exact mechanism of kidney failure. Our study revealed that AKI was present under the form of hepatorenal syndrome in eight cirrhotic patients. CKD was not found at all in the cirrhotic population. 

**Acknowledgement**

This paper was published under the frame of European Social Found, Human Resources Development Operational Programme 2007-2013, project no. POSDRU/159/1.5/S/133377.
